# Twin pregnancy outcome following teriflunomide treatment in a relapsing-remitting multiple sclerosis patient: A case report

**DOI:** 10.1097/MD.0000000000021212

**Published:** 2020-07-10

**Authors:** Giangaetano D’Aleo, Carmela Rifici, Antonina Donato, Francesco Corallo, Marcella Di Cara, Placido Bramanti, Edoardo Sessa

**Affiliations:** Istututo di Ricovero e Cura a Carattere Scientifico IRCCS Centro Neurolesi “Bonino-Pulejo”, Messina, Italy.

**Keywords:** pregnancy, relapsing-remitting multiple sclerosis, terifluonomide2233

## Abstract

**Rationale:**

Teriflunomide is a disease-modifying drug that has been approved for treatment of relapsing-remitting multiple sclerosis. Due to its teratogenic effect in animals, however, it is not recommended during pregnancy. For this reason, effective contraception must be used during its administration. When an unscheduled pregnancy occurs during therapy, patients must undergo a cholestyramine procedure for rapid flushing of the drug.

**Patient concerns:**

We describe the case of a 35-year-old female patient suffering diagnosed with relapsing-remitting multiple sclerosis at the age of 20. The patient as a result of side effects of previous therapies started taking teriflunomide.

**Diagnosis:**

Despite recommendations for the use of contraceptives, the patient became pregnant during drug therapy. Pregnancy occurred 12 months after initiating teriflunomide treatment.

**Interventions:**

Therapy with teriflunomide was immediately suspended and cholestyramine was prescribed (8 g 3 times a day, for 11 days) to flush out any residual drug from the body.

**Outcomes:**

Despite an 8-week exposure to teriflumomide during gestation, the patient gave birth to healthy twin girls at 35^th^ week. Controls carried out after birth did not reveal any malformation or genetic and chromosomal abnormality. At a 5-month pediatric specialist check both babies were healthy and growing regularly.

**Conclusion:**

This shows that even if there is evidence of teratogenic effects in animals, an 8-week exposure to teraflunomide >0.02 mg/L did not have effects on the newborn.

## 1 Introduction

Multiple scleroris (MS) should not be associated with an increased risk of adverse pregnancy outcome. However, prenatal exposure to disease-modifying therapies (DMTs) may have a potentially negative effect on fetal development.^[1–4]^

Teriflunomide is a daily oral immunomodulating disease-modifying drug approved for treatment of relapsing-remitting (RR) MS. It is not recommended during pregnancy due to observed teratogenicity and embryolethality in rat and rabbit offspring. In vitro studies instead showed no teriflunomide mutagenicity, whereas in vivo it was not clastogenic.^[5]^

Animal studies indicate a minimal teratogenic risk for the fetus if teriflunomide plasma concentration is <0.02 mg/L. However, to achieve values <0.02 mg/L a minimum of 8 months are needed up to a maximum of 2 years due to individual variability.

The use of effective contraception is very important. Women wishing a pregnancy should inform institution where teriflunomide will be discontinued for all the gestational period. If unprogrammed pregnancy should occur, the patient must undergo an accelerated elimination procedure using cholestyramine 8 g every 8 hours for 11 days, or activated charcoal (50 g every 12 hours). Once plasma teriflunomide concentration is <0.02 mg/L threshold, the test is repeated at a 14-day interval. If both plasma concentrations are <0.02 mg/L, no risks for the fetus are expected.^[5]^

However, despite recommendation for effective contraception, clinical trials have reported pregnancies after the approval of the drug by the regulatory agencies.

A retrospective analysis of the global pharmacovigilance database reported, at data cut-off, 70 pregnancies in female patients with teriflunomide treatment and 19 pregnancies in female partners of male patients. All newborns were healthy presenting no structural or functional abnormalities. Documented median birth weight was 3.3 kg in 18 newborns, whereas documented mean gestational age in 23 cases was 39 weeks (range 36–44 weeks). The spontaneous abortion rate (18.6%) was within general population range (17%–22%). No induced abortions presented defects or malformations. Maternal exposure to terifluonomide ranged from 12 days to 4.5 years.^[5]^

A twin pregnancy was reported in a woman with known teriflunomide exposure, but no details were presented.^[5]^

## 2 Case report

We describe the case of a 35-year-old female patient suffering with RRMS at the age of 20. The patient is an abstemious nonsmoker, with no hypertension, diabetes mellitus, nor psychiatric disorders. She was in therapy with 30 μg/weekly intramuscular interferon-beta-1a from the age of 20 to the age of 25 years. Due to the appearance of a relapse, characterized by paraparesis, therapy was modified to 22 IU 3 times a week of subcutaneous interferon-beta-1a (44 IU dosage was not tolerated). This therapy was interrupted at the age of 34 due to the appearance of an intense flu-like syndrome and extensive skin reactions at the site of injection. Terifluonomide was prescribed at a dosage of 14 mg/day. The patient was informed about the possible side effects and the need to use effective contraception due to the lack of scientific evidence regarding the nonteratogenicity of this drug in humans.

The patient, however, did not abide to contraceptive recommendation nor did she request it from her partner. This resulted in an unscheduled pregnancy after 12 months of teriflunomide therapy. Believing it was a simple delay of the menstrual cycle, she carried out the pregnancy test only after 1 week. Furthermore, she informed our Institute of the positive pregnancy results after a week. Therapy with teriflunomide was immediately suspended and cholestyramine was prescribed (8 g 3 times a day, for 11 days) to flush out any residual drug from the body. The first plasma dosage of cholestyramine was then performed and resulted >0.02 mg/L. Patient revealed that due to intense vomiting cholestyramine had not been taken regularly. Therapy with cholestyramine 8 g 3 times a day was therefore continued for another 11 days. Two other plasma dosage of cholestyramine were performed at the end of the second 11 days and after a further 2 weeks, with both plasma values of teriflunomide <0.01 mg/L.

The patient was again informed of the lack of scientific evidence of nonteratogenicity of the drug in humans. The patient decided to continue the pregnancy.

Patient was prescribed and took folic acid 400 μg/day during the first trimester and 800 μg/day in the second and third trimesters. At the 6th week of pregnancy, an ultrasound revealed the presence of a twin pregnancy. Later ultrasounds showed a single placenta and 2 amniotic sacs. Patient refused to undergo amniocentesis due to risk for fetuses. The course of gestation was regular. Periodic controls for rubella infections, toxoplasmosis, and cytomegalovirus were always negative. Birth occurred by cesarean section at the 35th week. Two baby girls were born, the first weighing 1990 g and the second 1940 g, both 43 cm long; both presented an Apgar index of 10/10 at 1 and 5 minutes. The ultrasound and genetic controls, carried out on the babies after birth, did not reveal any malformation or genetic or chromosomal abnormality.

Breast-feeding was carried out for 3 weeks then interrupted due to depletion of the milk supply. The menstrual cycle resumed 1 month after giving birth. The patient undertook treatment with teriflunomide again.

Pediatric follow-up of the babies confirmed regular growth and development (they weighed 5800 g and 5700 g, respectively and were both 52 cm in length).

No relapse was observed during pregnancy and the following 5 months after birthing (follow-up reported in this case).

## 3 Discussion

This case confirms that exposure to teriflunomide plasma levels >0.02 mg/L, for a period of a few weeks after conception, does not necessarily damage the embryo, both in terms of genetic malformation, organogenesis, and development. This is confirmed by the twin pregnancy in the case described. This is the second case mentioned in scientific literature, but it is certainly the most detailed.

Post-conception exposure to teriflunomide was calculated as 8 weeks: ovulation, 14 days pre-scheduled menstrual cycle, 7 days before the pregnancy test, 7 days before contacting our Center, 11 days of the first treatment with cholestyramine, 6 days before outcome test, followed by 11 days of treatment with cholestyramine, to obtain serum teriflunomide values <0.01 mg/L, for a total of 56 days.

The length of exposure to teriflunomide was due to nonprogrammer pregnancy and gravidarum hyperemesis that slowed the elimination procedure.

Although there was no damage to the fetus, the use contraceptive measures should be reinforced in patients treated with teriflunomide. However, for those who wish a pregnancy, there is no evidence as to the nonteratogenicity and fetal damage in humans. In the case of aggressive forms of the disease, other drugs should be used if the patient wishes a pregnancy.^[6]^ The case, assessed in the light of clinical risk management, raises the question of more personalized information regarding patients desire for pregnancy (cognitive level, culture, family context).

## Author contributions

**Conceptualization:** Francesco Corallo

**Data curation:** Marcella Di Cara

**Formal analysis:** Carmela Rifici

**Methodology:** Francesco Corallo

**Supervision:** Placido Bramanti

**Validation:** Edoardo Sessa

**Visualization:** Giangaetano D’Aleo.

**Writing – original draft:** Antonina Donato.
